# Real‐time polymerase chain reaction quantification of the salivary levels of cariogenic bacteria in patients with orthodontic fixed appliances

**DOI:** 10.1002/cre2.285

**Published:** 2020-03-17

**Authors:** Manal A. Al‐Melh, Radhika G. Bhardwaj, Eunice M. Pauline, Maribasappa Karched

**Affiliations:** ^1^ Department of Developmental and Preventive Sciences, Faculty of Dentistry Kuwait University Kuwait City Kuwait; ^2^ Oral Microbiology Research Laboratory, Department of Bioclinical Sciences, Faculty of Dentistry Kuwait University Kuwait City Kuwait

**Keywords:** orthodontic brackets, plaque, real‐time PCR, saliva, *Streptococcus mutans*, *Streptococcus salivarius*

## Abstract

**Aim:**

The aim was to investigate the salivary detection frequencies and quantities of caries‐associated bacteria from patients with orthodontic brackets.

**Methods:**

Patients wearing orthodontic brackets (*n* = 40, mean age = 26 years) and healthy controls without brackets (*n* = 40, mean age = 17 years) were enrolled in the study. Saliva samples from each patient was collected. After DNA purification, target species comprising streptococci and a *Lactobacillus* species were detected and quantified from the samples using polymerase chain reaction (PCR) and real‐time quantitative PCR.

**Results:**

Detection frequencies did not differ between the orthodontic patients and the control subjects for any target species except for *Streptococcus sobrinus*, which showed significantly lower detection rates in orthodontic patients (*p* < .05). *Lactobacillus casei* and *Streptococcus gordonii* were found at the highest detection frequencies with both species being detected in 38 (95%) of the saliva samples of orthodontic patients. Similarly, *L. casei* and *Streptococcus salivarius* were the species with highest detection frequencies (35, 87.5%) in the control subjects. Real‐time PCR revealed that *Streptococcus mutans* and *S. salivarius* quantities were significantly higher in orthodontic patients than in the control subjects (*p* < .05).

**Conclusions:**

Application of orthodontic brackets for 12 months leads to increased salivary levels of cariogenic bacteria and may serve as a potential risk factor for caries initiation.

## 1 INTRODUCTION

Accumulation and retention of dental plaque around orthodontic fixed appliances is a common problem (Freitas, Marquezan, Nojima Mda, Alviano, & Maia, 2014; Ristic, Vlahovic Svabic, Sasic, & Zelic, 2007). Subsequently, the plaque retained around the orthodontic fixed appliances can cause enamel demineralization and decalcification that can progress to carious lesions (Ahn, Lim, & Lee, 2007; Ahn, Lim, Yang, & Chang, 2005). Enamel demineralization usually occurs in areas around the orthodontic brackets due to dental plaque accumulation (Ahn, Kho, Lee, & Nahm, 2002; Mitchell, 1992).

There are also a few reports showing that fixed appliances might impede effective oral hygiene and cause an increased risk for caries initiation (Bretas, Macari, Elias, Ito, & Matsumoto, 2005; Turkkahraman et al., 2005). Furthermore, based on the difficulty of maintaining oral hygiene, the oral microbiota may also be influenced by orthodontic appliances (Naranjo, Trivino, Jaramillo, Betancourth, & Botero, 2006; Thornberg et al., 2009). These variables would possibly lead to microbial dysbiosis leading to a shift in increased colonization by pathogenic bacteria possessing potent virulence factors that contribute to gingival inflammation, periodontal support destruction (Naranjo et al., 2006; Thornberg et al., 2009), and changes in enamel surface (Bretas et al., 2005; Sukontapatipark, el‐Agroudi, Selliseth, Thunold, & Selvig, 2001; Turkkahraman et al., 2005). Further, there are a number of studies on orthodontic treatment and the risk for adverse effects, such as the development of white spot lesions (Khoroushi & Kachuie, 2017; Srivastava, Tikku, Khanna, & Sachan, 2013). Hence, the orthodontic patients are considered to be “at‐risk” patients.

There is considerable evidence in the literature, demonstrating that the presence of fixed orthodontic appliances in the oral cavity of dental patients could influence changes in oral microbial profiles (Freitas et al., 2014). Following the application of orthodontic appliances, the structure, metabolism, and composition of dental plaque would change, leading to a general increase in the levels of microbial population, particularly *Streptococcus* and *Lactobacillus* (Sukontapatipark et al., 2001).

Bacterial cells are continuously detached from dental plaque biofilms. Saliva constantly washes mucosal and dental surfaces and collects detached bacteria. Saliva with live bacteria (von Troil‐Linden, Torkko, Alaluusua, Jousimies‐Somer, & Asikainen, 1995) is the likely vehicle for intraoral but also person‐to‐person transmission of periodontitis‐associated bacteria, with their levels being a determinant for successful transmission (Asikainen, Alaluusua, & Saxen, 1991). Free planktonic bacteria cannot multiply in saliva since they get swallowed constantly, but after each swallowing incident part of whole saliva remains on oral surfaces (Lee, Crouse, & Kline, 2010), in this case, orthodontic appliances, which may result in reattachment of bacteria. Despite the development of new orthodontic appliances, new bonding techniques and material, it has not yet been possible to reduce dental plaque retention (Ristic et al., 2007). This study may provide important new information not only on the detection frequencies but also the absolute quantities of major cariogenic bacteria that colonize around Damon Q self‐ligating brackets. Eventually, this may help orthodontists reinforce proper oral hygiene measures for the orthodontic patients. Thus, the aim of this study was to assess the detection frequencies and quantities of species belonging to the genera *Streptococcus* and *Lactobacillus*, which are known to play a major role in dental caries, from the saliva samples of patients wearing orthodontic appliances.

## 2 MATERIAL AND METHODS

### 2.1 Ethical approval

All procedures performed in studies involving human participants were in accordance with the ethical standards of the institutional and/or national research committee and with the 1964 Helsinki declaration and its later amendments or comparable ethical standards. The study's experimental design and protocol were approved by the Ethical Committee of the Health Sciences Center of Kuwait University. The ethical approval was obtained on July 4th, 2017. A written informed consent was obtained from all the patients.

### 2.2 Study subjects

Forty patients (8 males, 32 females), with a mean age of 26 years (*SD*: 8.05), receiving orthodontic treatment with fixed orthodontic brackets were recruited to the study. The control group without orthodontic brackets consisted of 40 subjects (11 males, 29 females) with a mean age of 17 years (*SD*: 9.53). None of the approached patients declined to participate. The sample size was estimated based on previous literature and by using the online tool “ClinCalc” (https://clincalc.com/stats/samplesize.aspx). For a sample size that would allow us to detect the differences between the groups, a test power of 90% and *α* = 5% were considered.

The inclusion criteria involved patients with intact maxillary and mandibular teeth with fixed orthodontic appliances for at least 12 months. The fixed orthodontic appliances included Damon Q. 022 slot self‐ligating stainless steel brackets, which were placed on the labial surfaces of the maxillary and the mandibular permanent teeth up to the first molars, which received bondable tubes.

The steps for bracket bonding were the same for all patients receiving orthodontic treatment with fixed appliances. Prior to bonding, the teeth were cleaned using pumice paste, and then, the teeth were carefully washed and dried. Proper isolation was obtained with the use of cheek retractors, cotton rolls, and saliva ejectors.

First, the enamel of the teeth receiving the brackets was etched with 35% phosphoric acid (Ultra‐Etch) for 30 s. Then, the enamel surfaces were adequately rinsed with water for 10 s and thoroughly dried until the enamel appeared chalky white. After that a bonding agent was applied on the enamel surfaces and was gently dried (3 M Unitek), and the enamel surfaces were light cured for 10 s. Last, each bracket was bonded using Transbond Plus Light Cure Orthodontic Adhesive (3 M Unitek), and every bracket was light cured for 20 s.

Patients with gingival inflammation, missing teeth, active carious lesions, and prosthetic crowns were excluded from the study. Moreover, patients with systemic diseases, patients receiving systemic medication, and pregnant women were excluded from the study. All patients were given the same oral hygiene instructions at the start of the study.

### 2.3 Clinical parameters and sample collection

Plaque index scores from the orthodontic patients and the controls were recorded on a scale of 0–3 (Silness & Loe, 1964).

For collecting the saliva sample, each patient was given a paraffin wax pellet (Ivoclar Vivadent AG, Liechtenstein, Germany) to chew on while providing the saliva. While chewing on the wax pellet, the patient was asked to gradually expectorate the saliva in a sterile 50 ml Corning Falcon Conical Centrifuge Tube (Fisher Scientific) until 5 ml was obtained. Each falcon tube was labeled with the patient's serial number. The samples from each patient were placed in an ice box to be transported to the Oral Microbiology Research Laboratory, Faculty of Dentistry, at Kuwait University for the analyses.

### 2.4 Bacterial strains, media, and culture conditions

Reference bacterial strains *Streptococcus mutans* CCUG 11877 T, *Streptococcus salivarius* CCUG 50207, *Streptococcus sobrinus* CCUG 25735, *Streptococcus gordonii* CCUG 33482, and *Lactobacillus casei* OMG 3184 were cultured on brucella blood agar media plates containing 5% sheep blood and incubated at 37°C in 5% CO_2_ in air for 2 days.

### 2.5 Laboratory processing of samples

Samples collected were processed immediately or maximum within 24 hr of sampling. Saliva samples were centrifuged at 5000×*g* for 10 min, supernatants were discarded and the pellets were resuspended in 1 ml of phosphate buffered saline. The saliva samples were centrifuged at 18000×*g* for 5 min to recover pellets. Supernatants were discarded and the pellets were stored at −80°C until further testing.

### 2.6 DNA purification

Total DNA from the saliva samples and from the reference bacterial strains were purified using DNeasy Blood and Tissue Kit according to manufacturer's instructions (Qiagen). In brief, bacterial cells in the sample were lysed by incubating at 56°C for 1 hr in 180 μl of animal tissue lysis buffer and 20 μl of Proteinase‐K. DNA in the lysate was further precipitated in lysis buffer (200 μl) and absolute ethanol (200 μl).

The lysed sample was loaded on the membrane column provided with the kit and washed with wash buffers to remove contaminants and enzyme inhibitors, followed by centrifugation at 18000×*g*. Finally, the DNA bound to the membrane was eluted in 200 μl nuclease free water on centrifugation at 6000×*g* for 1 min. DNA concentration was measured by UV spectrometry method on NanoDrop 1000 (Thermo Scientific).

### 2.7 Validation of species‐specificity of 16S rDNA primers

The specificities of the primers for polymerase chain reaction (PCR) and quantitative PCR (qPCR) chosen from the literature were confirmed by in silico analysis (Table 2). Each primer sequence was run in NCBI BLAST 16S rRNA sequence database for bacteria and archaea. Further, the primer sequences were checked for specificity in the Human Oral Microbiome Database 16S rRNA RefSeq vs15.2 (The Forsyth Institute, Cambridge, MA). Finally, to verify the primer binding sites and estimated amplicon size in each of the 16S rDNA sequences of the test bacteria, open source bioinformatics tools such as Clustal Omega, an alignment tool, was used.

### 2.8 Polymerase chain reaction

Target bacterial genes in the purified DNA from saliva samples were amplified by PCR using 16 s rDNA species specific primers chosen from literature (Table 1) and their specificity revalidated using in silico analysis tools (Karched, Bhardwaj, Inbamani, & Asikainen, 2015). Following was the reaction mixture for PCR: 4 μl 5× FIREPol Master mix (Solis BioDyne), 0.5 μl each of forward and reverse primer (0.25 μM), 10 μl nuclease free water and 5 μl template. Amplifications were carried out on Veriti Thermal Cycler and Gene Amp PCR system 9,700 (Applied Biosystems) using the following thermal profile: Followed by a 10‐min initial denaturation at 95°C, 35 cycles of 95°C for 30 s^−1^ min, 50–63°C (depending on the primer pair) for 30 s and 72°C for 30 s^−1^ min. Amplification products were run on 2% agarose gels followed by ethidium bromide staining and the amplicons were further visualized and documented using imaging system (SynGene; G‐Box).

**Table 1:** Primers and annealing temperatures used in polymerase chain reaction (PCR) of five orthodontic bacterial species

Primer sequences 5′‐ 3′	Annealing temperature (°C)	Product size (bp)	References
***Streptococcus mutans*** F = TCGCGAAAAAGATAAACAAACA R = GCCCCTTCACAGTTGGTTAG	63	479	(Chen et al., 2007)
***Streptococcus salivarius*** F = GTGTTGCCACATCTTCACTCGCTTCGG R = GAGCAGCTACTCCAGCATTT	55	345	(Hoshino et al., 2004)
***S*** *treptococcus* ***sobrinus*** F = GAGTTTGATCCTGGCTCAG R = TTAGTAACACTGGAGCAAGTCCG	50	95	(Rupf, Merte, Eschrich, Stosser, & Kneist, 2001; Sakamoto, Takeuchi, Umeda, Ishikawa, & Benno, 2001)
***Streptococcus gordonii*** F = GGTGTTGTTTGACCCGTTCAG R = AGTCCATCCCACGAGCACAG	53	98	(Suzuki, Nakano, Yoshida, Yamashita, & Kiyoura, 2004)
***Lactobacillus casei*** F = TTAAAGCCATTCTCAGTTCGGA R = TACGGCTACCTTGTTACGACTT	55	232	(Sakamoto et al., 2001; Ward & Timmins, 1999)

### 2.9 Real‐time qPCR

Absolute quantification of the levels of *S. mutans* and *S. salivarius* was achieved by real‐time PCR (RT‐PCR). For qPCR quantification of the six periodontal species, previously validated species‐specific primers for 16S rRNA gene (Table 2) were chosen and their specificity revalidated using in silico analysis tools (Karched et al., 2015). The following reaction mixture was used: 10 μl SYBR Green master mix (Power SYBR Green Kit, Applied Biosystems), 0.4 μl each of forward and reverse primer (0.2 μM), 7.2 μl H_2_O and 2 μl DNA template (containing at least 20 ng DNA).

**Table 2:** In silico validation of the specificity of the primers using Human Oral Microbiome Database for 16S rRNA gene sequences and NCBI BLAST

Species	No. of forward primer binding sites; specificity	No. of reverse primer binding sites; specificity
*Streptococcus mutans*	1; *S. mutans*	2; *S. mutans* and *S. pyogenes*
*Streptococcus gordonii*	1; *S. gordonii*	1; *S. gordonii*
*Streptococcus sobrinus*	Universal primer	1; *S. sobrinus*
*Streptococcus salivarius*	1; *S. salivarius*	1; *S. salivarius gtf*K gene But may potentially bind to other regions in the genome. **Note:** F primer specific to *S. salivarius*
*Lactobacillus casei*	2; *L. casei* and *Lactobacillus paracasei*	Universal

*Note*: Number of primer binding sites and species specificity are shown. Primer sequences and references presented in Table 1.

Reaction was carried out on ABI Fast RT‐PCR machine using the following thermal cycling profile: After a 10‐min initial denaturation at 95°C, 40 cycles of 95°C for 15 s, 55°C for 30 s, and 72°C for 30 s were run. Data was analyzed using the software SDS 1.4.0v. Serial dilutions of DNA from the above two streptococcal species were used in the reaction and the Ct values were plotted against deduced bacterial cell concentration (cells/ml) for each species, to construct standard curves using the above software. The acceptable reaction efficiency was set at 86–105% (slope −3.7 to −3.2) and *R* ^2^ value for standard curve linearity was 0.97–0.99.

### 2.10 Statistics

Differences in bacteria detection frequencies between the groups were determined using McNemar test. A *p* value of <.05 was considered statistically significant.

Bacterial counts were log10 transformed after adding 1 to all data to handle zero counts. Normality of the data was tested by Skewness and Kurtosis values, Shapiro Wilkins *p* values, and histograms. Since the data did not comply with normal distribution, a nonparametric Mann–Whitney *U* test was used to compare differences between groups.

Two independent PCR and Real‐Time PCR experiments were carried out for all samples. The statistical software SPSS version 25.0 (IBM Corp, Armonk, NY) was used for all analyses.

## 3 RESULTS

The plaque index scores from the patient group ranged between 2 and 3, while the scores for healthy subjects were 0–1.

**Figure 1:**
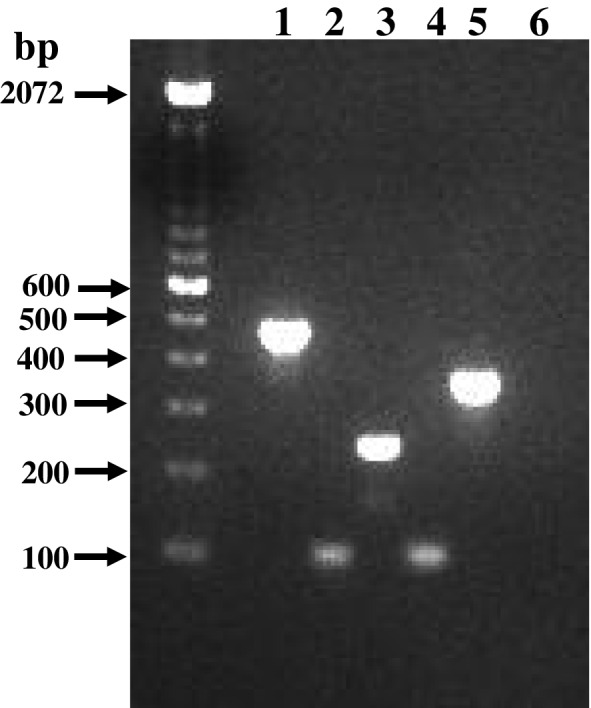
Agarose gel showing polymerase chain reaction (PCR) amplicons from *Streptococcus* and *Lactobacillus* species in patients with orthodontic fixed appliances. PCR was performed using species‐specific primers and the PCR amplicons were separated on a 2% agarose gel. Lanes: M, 100 bp marker; Sm, *Streptococcus mutans*; Ssob, *Streptococcus sobrinus*; Lc, *Lactobacillus casei*; S sal, *Streptococcus salivarius*; Sg, *Streptococcus gordonii*; Neg, negative control; MW, molecular weight; bp, base pair

PCR amplification of 16S rRNA genes from the reference strains of the target species showed bands of expected sizes deduced from the respective DNA sequences (Figure 1 and Table 1). All reference strains produced single bands confirming the absence of nonspecific amplification.

Table 3 presents the detection frequencies of all target species from the patients receiving fixed orthodontic brackets and control subjects with no orthodontic treatment. Except for *S. sobrinus* (*p* < .05), no significant difference was seen in the detection frequencies between orthodontic patients and control subjects. *Lactobacillus casei* and *S. gordonii* were found at the highest detection frequencies with both species being detected in 38 (95%) of the saliva samples. The next species in decreasing order of detection rates were *S. salivarius*, 33 (82.5%); *S. mutans*, 28 (70%) and *S. sobrinus* 5 (12%).

**Table 3:** Polymerase chain reaction (PCR) detection frequencies of target bacterial species in saliva samples from orthodontic patients and controls

	Species detection frequency (*N* = 40 subjects)				
	*Streptococcus mutans*	*Streptococcus salivarius*	*Streptococcus sobrinus*	*Streptococcus gordonii*	*Lactobacillus casei*
Orthodontic	28 (70%)	33 (82.5%)	5 (12%)	38 (95%)	38 (95%)
Controls	31 (77.5%)	35 (87.5%)	36 (90%)	32 (80%)	35 (87.5%)

In the case of control subjects, *L. casei*, similar to its detection frequency in orthodontic patients, was the most detected species (35, 87.5%) together with *S. salivarius* (35, 87.5%). The detection frequencies of the other species were for *S. gordonii*, 32 (80%); *S. mutans, 31 (77.5%)*, and *S. sobrinus* 27 (67%).

Bacterial detection frequencies from saliva samples differed significantly (*p* < .05) between the target species with the exception that *S. salivarius* detection rates did not differ from those of *S. mutans*, *S. gordonii*, and *L. casei* (*p* > .05). Similarly, detection rates between *L. casei* and *S. gordonii* did not show significant difference.

**Figure 2:**
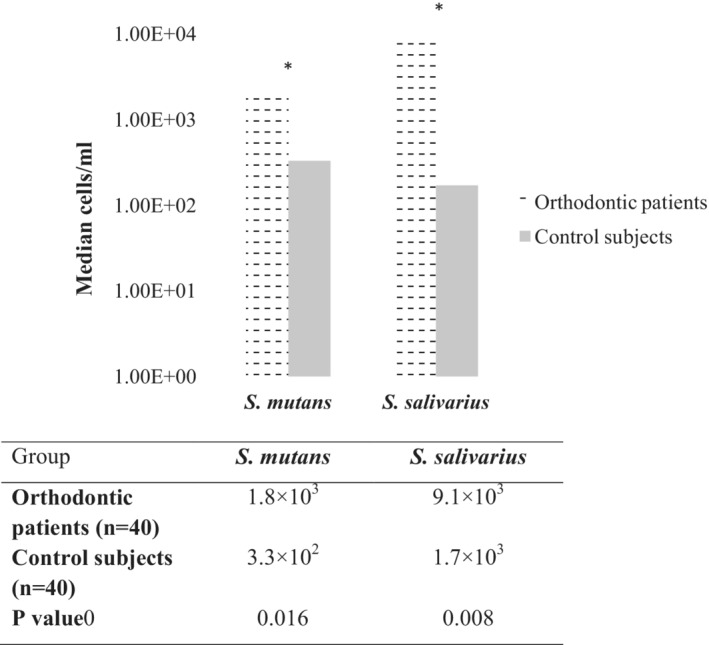
Quantities of cariogenic bacteria in the saliva samples of orthodontic patients and control subjects, as determined by real‐time polymerase chain reaction (PCR). Purified DNA from the patient samples were analyzed by SYBR‐Green quantitative PCR (qPCR) using species‐specific primers. Bacterial quantities are presented as median cell numbers from all subjects in each group. *p < .05

Since no significant differences in the detection frequencies between the orthodontic patients and the controls were found, we next sought to determine quantities of the target species in the study group and the control group. For this purpose, we chose *S. mutans* and *S. salivarius*, two of the most important species in dental caries. As quantified by the real‐time PCR method (Figure 2), both *S. mutans* and *S. salivarius* were in significantly higher quantities in orthodontic patients than in control subjects (*p* < .05). The median cells per ml for *S. mutans* were 1.8 × 10^3^ (ortho patients) and 3.3 × 10^2^ (controls). Similarly, for *S. salivarius*, 9.1 × 10^3^ (ortho patients) and 1.7 × 10^3^ (controls).

## 4 DISCUSSION

While detection frequencies of the target streptococci and *Lactobacillus* species generally did not differ significantly between the controls and the orthodontic patients, real‐time qPCR revealed that quantities of *S. mutans* and *S. salivarius* were significantly higher in orthodontic patients than in control subjects. In oral infections, which are polymicrobial in nature, it is alterations in quantities of specific bacterial species, not mere presence or absence that indicates dysbiosis and sets up initiation of infection process.

Primer specificity is critical in getting accurate information of the quantities of target species from clinical samples. Although we chose the primers from literature based on their reported specificity for target species, we confirmed the specificity in silico using bioinformatics tools and databases. Each primer pair was found to be species‐specific except for *L. casei*, in which, the forward primer had a binding site in *Lactobacillus paracasei* as well. Therefore, while detection/quantification of *L. casei* using the present primer pair in this study must be considered cautiously, new primer pair with absolute specificity for *L. casei* need to be optimized in future studies.

Several studies examined the association between orthodontic appliances and oral infections such as caries. While a number of longitudinal studies have reported a significant increase in the counts of major oral bacteria associated with caries or periodontitis (Maret et al., 2014; Pan et al., 2017), the aim of this study was to investigate the occurrence frequencies and quantities of select cariogenic bacterial species in saliva from patients wearing orthodontic fixed brackets in Kuwait.

In the current study, we found that both *S. mutans* and *S. salivarius* were in significantly higher quantities in orthodontic patients than in control subjects. It is plausible that plaque accumulated around orthodontic appliance is the source of elevated levels of these bacterial species in the saliva. In fact, it has been long known that free planktonic cells are continuously detached from mature biofilms (Costerton et al., 2003). The biofilm‐derived bacterial cells may in turn attach to new surfaces on the orthodontic appliances leading to formation of new plaque biofilm. This cyclic process of free cell detachment from plaque, reattachment to new surfaces and formation of new plaque becomes exacerbated when there is host‐microbe dysbiosis, eventually leading to initiation of oral infections.

Different studies have reported contradictory results on whether orthodontic treatment with brackets leads to caries development (Mattousch, van der Veen, & Zentner, 2007; van der Veen, Attin, Schwestka‐Polly, & Wiechmann, 2010). It was previously demonstrated that the irregular surfaces of the orthodontic fixed appliances contribute to the increase in growth of the *S. mutans* (Chang, Walsh, & Freer, 1999).

Several studies have investigated the presence of microbial species in plaque or saliva after a certain period of time (Kim et al., 2012; Maret et al., 2014; Pan et al., 2017; Topaloglu‐Ak, Ertugrul, Eden, Ates, & Bulut, 2011). In our study, target species were detected in saliva after 12 months of orthodontic therapy with fixed appliances. In contrast to our study showing elevated salivary quantities of *S. mutans* and *S. salivarius* in orthodontic patients compared to controls, a previous study found that some *Pseudomonas* species, but not streptococci, were found in increased numbers in orthodontic patients (Sun, Ahmed, Wang, Dong, & Niu, 2018). Although the authors suggested that the observed differences were because of better adherence capabilities of *Pseudomonas* compared to streptococci, numerous studies have shown streptococcal species to possess avid surface attachment properties (Takahashi, Urano‐Tashiro, & Konishi, 2013).

Increased occurrence and quantities of cariogenic streptococcal species following the application of orthodontic appliances has been well‐documented. As discussed elsewhere (Ristic et al., 2007), perhaps it is imperative that this is due to plaque retention caused by orthodontic appliances. It is important to clarify that plaque retention in patients wearing orthodontic appliances is not because of poor oral hygiene practice, but rather due to inappropriate or incomplete oral hygiene procedures. Higher salivary levels of cariogenic streptococci warrants better oral hygiene approaches, for example, more efficient mouth wash liquids to diminish salivary bacterial loads.

Another study assessed the microbial changes in the saliva of children with fixed orthodontic appliances compared with a control group of children without orthodontic treatment. Although this study was conducted on children, it was demonstrated that wearing a fixed orthodontic appliance for 6 months was associated with high levels of *S. mutans* and *Lactobacillus* species (Maret et al., 2014). This finding was in agreement with our study, which showed that there is an increase in *S. mutans* and S. *salivarius* after 12 months of treatment with fixed orthodontic appliances. There is evidence in the literature that the presence of fixed appliances influences the quantity and quality of oral microbiota (Lucchese, Bondemark, Marcolina, & Manuelli, 2018). Moreover, orthodontic treatment alters the oral environmental factors, leading to an increase in stimulated flow rate, buffer capacity, and salivary pH, which can affect the anti caries activity of saliva (Chang et al., 1999). Therefore, it seems necessary to evaluate the microbial parameters and stress oral hygiene preventive measures until the termination of orthodontic treatment.

An important limitation of this study was that we investigated only a few bacterial species of importance in caries development and progression. With the increasing utility of NextGen sequencing technologies that reveal total microbiota of the samples in question, several novel bacterial species whose role in caries was hitherto unknown have been uncovered during the recent years. Sequencing techniques like 16S rRNA gene metagenomics would have provided a comprehensive delineation of the microbiota in the dental plaque retained around the orthodontic braces.

## 5 CONCLUSIONS

Significantly higher levels of cariogenic streptococci in the saliva of our orthodontic patients, suggests that patients should be prescribed to regularly use mouth washes that are clinically proven to be efficient not only in reducing plaque accumulation but also in controlling salivary levels of cariogenic bacteria.

## CONFLICT OF INTEREST

The authors declare that they have no conflict of interest.
